# Contamination of dental simulation rooms with mycobacteria

**DOI:** 10.1186/s12866-026-05428-3

**Published:** 2026-07-28

**Authors:** Anna M. Oertel, Stefan Rupf, Matthias Hannig, Alexander Halfmann, Gudrun Wagenpfeil, Uwe Schlotthauer, Madline P. Gund

**Affiliations:** 1 Clinic of Operative Dentistry, Periodontology and Preventive Dentistry, Saarland University Medical Centre, Kirrberger Str. 100, Building 73, Homburg, D-66421 Germany; 2 Synoptic Dentistry, Saarland University, Homburg, Germany; 3 Institute of Medical Microbiology and Hygiene, Saarland University Medical Centre, Homburg, 66421 Germany; 4 Institute of Medical Biometry, Epidemiology and Medical Informatics, Saarland University, Homburg, Germany

**Keywords:** Dental unit waterlines, Dental simulation units, Non-tuberculous mycobacteria, *M. gordonae*, *M. chelonae*, *M. chimaera*, Biofilms

## Abstract

**Background:**

Dental unit waterlines (DUWLs) often provide optimal conditions for pathogens, including non-tuberculous mycobacteria, causing various infections in patients and practitioners. Preventive measures for non-tuberculous mycobacteria (NTM) remain unclear. Dental simulation units, which are used for student training, are very similar to classic dental units. While the water quality of dental units has been the subject of numerous studies, dental simulation units have received little attention to date. In particular, there are very few studies on the role of NTM in dental simulation units and on influencing factors such as water stagnation, structural conditions, and the effectiveness of a disinfection program.

**Methods:**

In this study, 24 dental simulation units from two rooms in two different buildings of the Department for Operative Dentistry, Periodontology and Preventive Dentistry were examined for NTM. Water samples were analyzed from all simulation units, and biofilm swabs were additionally examined from room 2. All samples were incubated on solid and liquid culture media for 8 weeks. The species of NTM were identified using GenoType line probe assays.

**Results:**

NTM were detected in 43 of 44 samples. Three different species were identified: *M. gordonae* was the most common, with 24 detections, followed by *M. chelonae* with 17 detections and *M. chimaera* with 3 detections.

**Conclusion:**

The high contamination rate of dental simulation units highlights the need for further research to ensure a safe environment. No significant differences in terms of structural conditions could be found, while the influence of water stagnation and disinfection protocols warrants further investigation.

## Introduction

Dental units for clinical use are essential for dental treatment. However, they also pose a risk of infection for patients and practitioners, particularly through their water lines [1]. Dental unit waterlines (DUWL) are usually connected to the drinking water supply and carry water via their hose system to the motors and turbines of the treatment chair. There, the water is converted into an aerosol during treatment. The drinking water fed into the DUWLs is usually subject to microbiological testing, so the bacterial load is relatively low. In Germany, the microbiological contamination is regulated by the Drinking Water Ordinance, which stipulates a maximum bacterial load of 100 colony-forming units per milliliter (cfu/ml) [2]. Once drinking water enters dental units, it is no longer subject to the guidelines of the Drinking Water Ordinance, it then becomes process water [3]. However, according to the German Working Group for Hygiene in Dentistry, the number of colony-forming units should be a maximum of 100 cfu per milliliter, just as in drinking water [3].

Inside the DUWLs is a tubing system, which is typically made of silicone having a small diameter and, consequently, a large surface area. This leads to biofilm formation and further microbiological contamination [1, 4]. To counteract this, DUWLs are now equipped with disinfection systems. Particular attention is paid to contamination with *Legionella pneumophila* [5] and *Pseudomonas aeruginosa* [6], but there are several other potentially human pathogens. These include nontuberculous mycobacteria (NTM), which, according to a background document on the guidelines for drinking water quality [7], have become increasingly significant in recent years. Non-tuberculous mycobacteria are ubiquitous in the environment, especially in aqueous environments [8]. However, they can also cause infections, especially in immunocompromised individuals. Furthermore, Carson et al. reported that mycobacteria can also multiply in distilled water and may be resistant to various disinfectants [9]. Although guidelines and recommendations exist for contamination by Legionella pneumophila and Pseudomonas aeruginosa, contamination by mycobacteria has not yet been systematically investigated. Therefore, there are currently no recommendations for infection prevention.

Non-tuberculous mycobacteria are divided into fast-growing and slow-growing species. Fast-growing mycobacteria include, for example, *M. chelonae*, *M. fortuitum*, *M. abscessus* ssp. abscessus and *M. abscessus* spp. bolettii. Slow-growing mycobacteria include *M. gordonae*, *M. chimaera*, *M. interjectum*, *M. marinum*, and among others [8].

The different species of NTM also have different levels of pathogenicity. While *M. gordonae* is generally classified as low pathogenic [8, 10], *M. chimaera* has gained attention in recent years due to an outbreak following heart surgery. Contaminated heating and cooling units (HCUs) in operating rooms have been identified as a source of infections in the deep chest region, from which *M. chimaera* spreads via aerosols [11]. In 2024, Heinz et al. investigated mycobacterial contamination of dental units intended for clinical use at Saarland University Medical Centre. They found that 83% of the dental units examined were contaminated [12]. The present study examined dental simulation units at Saarland University Medical Centre. The simulation units are very similar in design to the dental units used in clinical practice with comparable DUWLs. However, unlike clinical dental units, they do not have a disinfection program. In addition, they are used exclusively by dental students meaning they are used much less frequently. Although the DUWLS of clinical dental units are not subject to guidelines for handling mycobacteria, several studies on mycobacterial contamination have already been conducted. The situation is different for simulation units, where no systematic studies on mycobacteria can be found in the current literature. Given their relevance from a hygiene and clinical perspective - particularly for immunocompromised individuals - an exploratory analysis appears warranted.

The aim of this study was to investigate mycobacterial contamination in dental treatment units without the use of disinfectants, as well as the effect of water stagnation on mycobacterial contamination of the DUWLs. In addition, the potential influence of structural factors should be investigated, such as whether the age of the building or its water pipes plays a role in mycobacterial contamination.

## Materials and methods

### Setting

For this study, two different student simulation rooms in the Department for Operative Dentistry, Periodontology and Preventive Dentistry at Saarland University Hospital were examined. Room 1 is located on the second floor of the Department for Operative Dentistry, a building that was last extensively renovated in 1984. The dental simulation units were newly installed in 2007. Room 2 is located in a separate small single-floor building, which was converted for this purpose in approximately 2014. The dental simulation units were purchased together with those from Room 1 in 2007 and were previously located at a different site. There is a total of 24 simulation units, 15 of which are located in Room 1 and 9 in Room 2. The simulation units are all connected to the municipal drinking water.

### Sampling

Water samples were collected from the air-water syringe of all 24 simulation units, and in both rooms, an additional water sample was taken from the regular faucet. For the samples from the air-water syringe, the syringe was first unscrewed, and then the sample could be taken directly from the supply hose. The tap aerator was dismantled before the samples were taken from the tap. The units were not flushed prior to sampling. All water samples were collected in a sterile 250 ml container. Of the 250 ml, approximately 50 ml was used for testing for mycobacteria. These containers were treated with sodium thiosulfate (20 mg/L, LP Italiana, Milan, Italy), which neutralizes any possible residues of disinfectants. All samples were taken by the same person. The subsequent examination for mycobacteria was carried out by microbiological specialists. In room 2 biofilm swabs (eSwab. Copan Diagnostics, Brescia, Italy) were taken and additionally tested for mycobacterial contamination. Due to capacity constraints in microbiological analysis, no swabs from room 1 were examined. The swabs were taken from the water-carrying system of the 9 simulation units. One silicone hose and the water inlet were sampled per unit. The silicone hose was tested over a length of 5 cm, and the water inlet was tested over its entire depth of 3.5 cm.

### Microbial cultivation

First, all samples were examined microscopically using auramine staining. Subsequently, both liquid and solid cultures were used for microbiological analysis. All culture media were checked for sterility and functionality in accordance with routine quality control procedures. The liquid culture tubes on Middlebrook 7H11 agar were utilized to examine broth-based mycobacterial growth, for which the Mycobacteria growth indicator tube (BACTEC MGIT 960 incubator, Becton Dickinson, Heidelberg, Germany) was used. Growth on solid culture was analyzed on Loewenstein-Jensen and Stonebrink Agar (Becton and Dickinson, Heidelberg, Germany). All samples were incubated for a period of 8 weeks. If mycobacterial growth was detected, species diagnosis was performed using different polymerase chain reaction assays (GenoType NTM-DR, Hain Lifescience, Nehren, Germany/ GenoType CM, GenoType AS).

### Statistical analysis

Differences in the detection frequencies of mycobacterial species between water samples from Room 1 and Room 2 were analysed using two-sided Fisher’s exact tests because of the small sample sizes. In Room 2, paired comparisons between water samples and the corresponding biofilm samples from the same simulation unit were performed using the exact McNemar test. A biofilm sample was considered positive if the respective mycobacterial species was detected in either the water inlet or the silicone hose swab. A p-value < 0.05 was considered statistically significant.

## Results

**Fig. 1:**
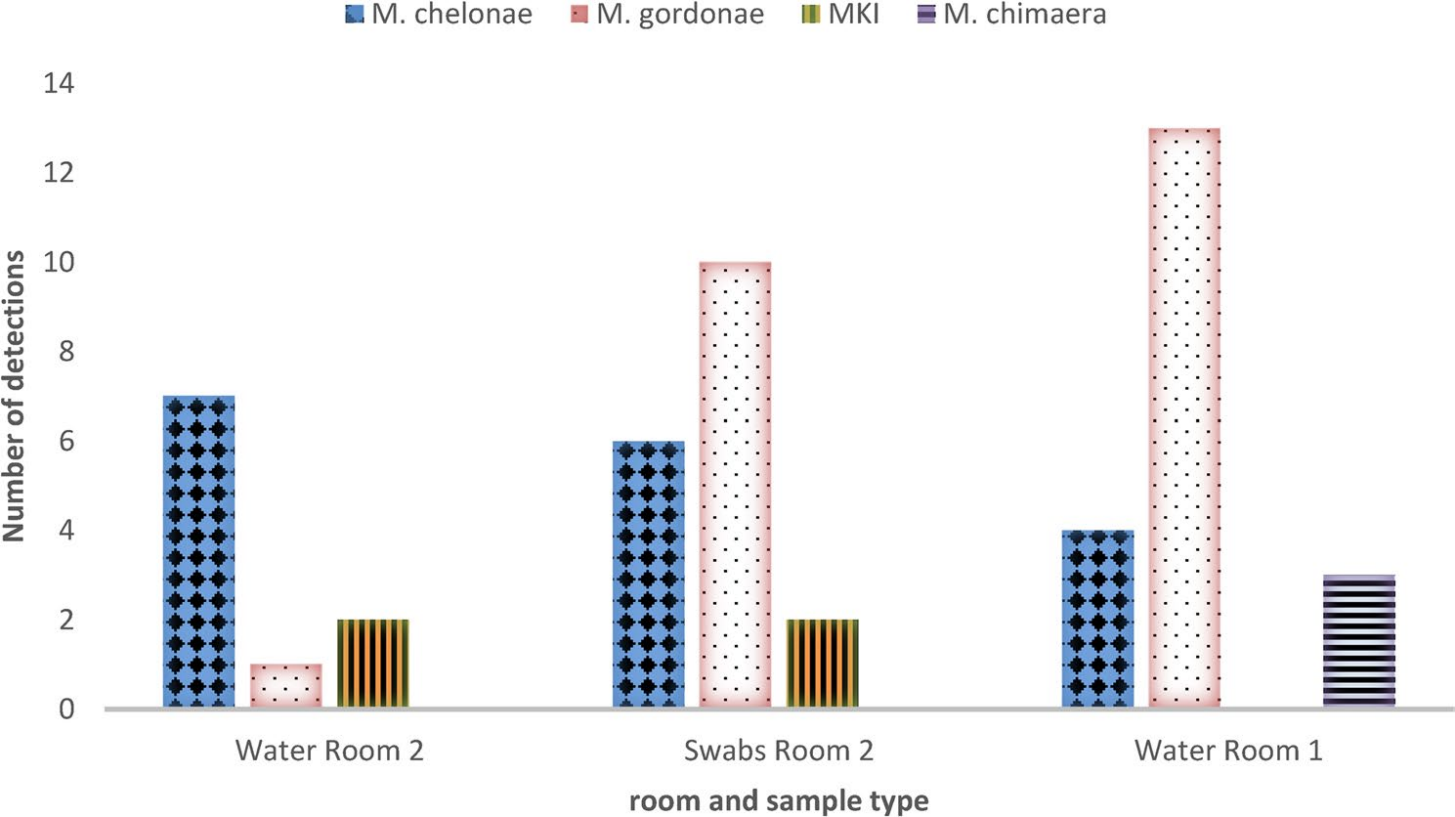
Number of mycobacteria species detected according to room and sample type

Mycobacteria were found in 43 of 44 samples with a total number of 48 detections. Three different species were detected, *M. gordonae*, *M. chelonae* and *M. chimaera*. *M. gordonae* was detected 24 times, *M. chelonae* 17 times, and *M. chimaera* 3 times. In four cases, it was not possible to make an accurate species identification. If a species cannot be identified, the result is reported as “mixed culture – unidentifiable” (MKI). Samples from both regular taps and 23 out of 24 simulation units were tested positive for mycobacteria. The results are shown in (Fig. 1; Table 1). Overall contamination rates between water samples from Room 1 and Room 2 did not differ significantly (*p* = 1.000).

**Table 1:** Representation of analyzed samples and positive results

	Water Room 1	Water Room 2	Swabs Room 2
Number of samples taken	16	10	18
Number of simulation units sampled	15	9	9
Number of regular taps sampled	1	1	/
Positive samples / simulation units (%)	93.75%	100%	100%
Number of samples containing more than one Species	5	0	0
Mycobacterial species identification	*M. gordonae* (13) *M. chelonae* (4) *M. chimaera* (3)	*M. gordonae* (1) *M. chelonae* (7) MKI (2)	*M. gordonae* (10) *M. chelonae* (6) MKI (2)

The water samples from room 1 tested positive 20 times, mycobacteria were detected in 15 of 16 samples, corresponding to a contamination rate of 93.75%. All 20 detections resulted in accurate species identification, and in five samples two different species were identified. All three different mycobacteria were found in the water samples of room 1, *M. gordonae* was identified 13 times, *M. chelonae* four times and *M. chimaera* three times. The five samples containing two different species of mycobacteria included combinations of the three different species. *M. gordonae* was detected three times together with *M. chelonae*, while *M. chimaera*/*M. gordonae* and *M. chimaera/M. chelonae* were detected once each. Compared with water samples from Room 2, *M. gordonae* was detected significantly more frequently in Room 1 (*p* < 0.001), whereas *M. chelonae* was detected significantly more frequently in Room 2 (*p* = 0.043). No statistically significant differences were observed for *M. chimaera* (*p* = 0.262) or mixed cultures (*p* = 0.138).

The water samples from room 2 tested positive 10 times, mycobacteria were detected in all 10 samples taken resulting in a contamination rate of 100%. In two cases, it was not possible to identify the species precisely. Only two different species of mycobacteria were found in the water samples from room 2: *M. gordonae* and *M. chelonae. M. gordonae* was identified one time, while *M. chelonae* was found 7 times.

The biofilm swabs from room 2 showed a contamination rate of 100% as well, with all 18 samples showing mycobacterial growth. Species identification could not be achieved in two samples. As in the corresponding water samples, only two different species of mycobacteria were identified. *M. gordonae* was detected ten times and *M. chelonae* six times.

Paired comparisons between water samples and the corresponding combined biofilm results from the nine simulation units in Room 2 showed no significant difference in the detection frequency of *M. chelonae* (*p* = 1.000). In contrast, *M. gordonae* was detected significantly more frequently in the combined biofilm samples than in the corresponding water samples (*p* = 0.039).

## Discussion

The aim of the study was to investigate mycobacterial contamination in two dental simulation rooms, with a particular focus on the impact of different structural conditions, the effect of prolonged periods of stagnant water, and the absence of a disinfection program.

In the present study, mycobacteria were detected in 43 of 44 water samples and biofilm swabs. Three different species were detected. *M. gordonae* was identified most frequently, with a total of 24 detections, followed by *M. chelonae* with 17 and *M. chimaera* with 3 detections. In four cases, species diagnosis could not be performed. *M. gordonae* and *M. chelonae* were found in the water samples from both rooms and in the biofilm swabs from room 2. *M. chimaera* was detected exclusively in the water samples from room 1.

It is well known that NTM are ubiquitous in the environment, especially in water systems and drinking water [13]. The NTMs enter the simulation units via the drinking water supply, where they are distributed via dental unit waterlines. The water-carrying elements of the dental simulation units consist of thin silicone tubes, which are characterized by a large surface area and a slow flow rate [4]. Combined with prolonged periods of standing water and a shortage of disinfectants, these conditions are ideal for the formation of biofilms within the simulation units.

NTM are known for their ability to form biofilms and resistance towards disinfectants [13]. Biofilms are organized, three-dimensional accumulations of bacteria and the extracellular polymers they produce at the interfaces of various materials. In the simulation units, they are present in liquid medium on the solid wall of the tubes [1]. Once a biofilm has formed, it provides a well-protected habitat for a variety of pathogens, including NTM.

Water samples were collected from the simulation units to assess the planktonic NTM load. To evaluate the presence of NTM within the biofilm, swabs were also taken in Room 2. Only qualitative contamination with NTM was examined, but not quantitative contamination such as concentration measurements. Consequently, the exact contamination rate of the individual samples could not be determined. Furthermore, without measuring the microbial load of NTM, no conclusions can be drawn regarding microbial exposure or its clinical and hygiene-related significance.

NTM can lead to infections, particularly in immunocompromised patients [8]. Nevertheless, cases in immunocompetent patients have also been reported [10]. The three identified NTM species are associated with various diseases, some of which can be severe.

The simulation units are used by dental students, who come into direct contact with the aerosols generated during use. The transmission of mycobacteria via aerosols is already described, Sax et al. reported in 2015 that *M. chimaera* from heater-cooler units (HCUs) infected patients via aerosols during heart surgery [11]. The infections described include wound infections, pulmonary infections, disseminated infections, and infective endocarditis [14]. *M. chimaera* is a slow-growing mycobacterium and was only characterized as a separate species in 2004 by Tortoli et al. [15].

Open-heart surgery cannot be directly compared to the situation in this study, but aerosolized mycobacteria can also cause lung infections. According to Schönfeld et al. [8], the incidence of NTM-related lung diseases has risen dramatically in recent decades. Respiratory tract infections caused by inhalation of *M. chimaera* and *M. gordonae* have been reported in immunocompromised and immunocompetent patients [10, 16–18]. *M. gordonae*, which is generally considered to be of low pathogenicity, is also classified as a slow-growing NTM. In addition to lung infections, infections of the skin and soft tissues as well as mastitis have been reported [19, 20].

*M. chelonae* is a fast-growing NTM that is frequently associated with soft tissue and skin infections, as well as catheter-related infections [21]. There are also case reports of eye infections, chronic lung disease, and disseminated infections [22].

Based on current data, infections with the identified NTMs cannot be ruled out even in immunocompetent individuals. Nevertheless, evidence shows that immunocompromised individuals are the group most at risk from mycobacteria. Since the immune status of the students using the simulation units is unknown, their individual risk cannot be assessed. In addition to students, cleaning staff, faculty, and technicians also come into contact with the water in the simulation units, which is why it must generally be assumed that potentially immunocompromised individuals may be at risk. Of the many possible infections caused by mycobacteria, we are particularly interested in those that affect the respiratory tract. As described above, all three detected species can cause respiratory tract infections. Whether the formation of aerosols at the simulation units could increase the risk of infection through inhalation cannot be assessed due to the study design. To draw any conclusions, quantification of NTM in the generated aerosols would be required.

Furthermore, this uncertainty underscores the importance of using personal protective equipment (PPE) correctly even in everyday, non-clinical settings. Several studies have already described how contamination can be reduced by wearing protective gloves and a face mask, particularly FFP-2 masks [23, 24].

In 2024, Heinz et al. conducted a study on dental treatment units for clinical use at the same hospital [12]. They examined the microbiological contamination of the treatment units, particularly with regard to NTM, which were detected in 35 of 42 water samples. Compared to the water sample results obtained in the present study, 25 of which tested positive out of 26, this indicates a slightly higher level of contamination in the dental simulation units. The types of bacteria detected in the simulation units were also found in the dental units intended for clinical use. In addition, two other species were detected in the dental treatment units. Although the general design of simulation units and clinical units are very similar, there are still some key differences. In particular, the significantly longer duration of use of the clinical units must be considered here. This longer duration of use also results in a larger flushing volume, which is further increased by the flushing protocol integrated into the units for clinical use. In addition, a disinfectant is added to the clinical dental units.

Therefore, the study design does not allow for direct causal conclusions regarding the higher contamination rate in the simulation units. Factors such as long stagnation periods - which are known to promote contamination [1, 25] - or the disinfection protocol may have had an influence on this. When selecting a disinfection protocol, special attention should also be paid to the known resistance of NTM to disinfectants [9]. To substantiate these hypotheses, further studies would be needed.

There were only minor differences between the two Simulation rooms in terms of their structural conditions. Samples from both rooms showed a high rate of mycobacterial contamination, particularly with *M. gordonae* and *M. chelonae*. In Room 1, which is located in a significantly older building with older water pipes, contamination with *M. chimaera* was also detected in three samples. In Room 2, which is about 30 years newer, no such contamination was detected. Due to the relatively small sample size and observational study design, this study cannot determine whether this is related to the varying ages of the buildings and water pipes. Further studies should be conducted to investigate the influence of structural conditions.

## Limitations

The study conducted is subject to different limitations. Species diagnostics could not be performed in all samples, resulting in limited comparability. Furthermore, the study design was qualitative only and no quantitative measurement of NTM was performed. Without knowing the quantity of NTM, it is not possible to assess the actual NTM exposure or a potential clinical risk.

## Conclusions

In this study, a high rate of contamination with mycobacteria was found both in the water and in potential biofilm from dental simulation units. All three NTM species detected are potentially pathogenic to humans, which is why an individual risk cannot be ruled out, particularly regarding immunocompromised individuals. Since only a qualitative analysis was performed, no conclusions can be drawn regarding the exact concentration and, consequently, the clinical relevance. The high number of NTM detected in the dental simulation units indicates that regular quality controls may be advisable to ensure a safe environment.

## Data Availability

The datasets used and analysed during the current study are available from the corresponding author on reasonable request.
